# Estimating metabolic equivalents for activities in daily life using acceleration and heart rate in wearable devices

**DOI:** 10.1186/s12938-018-0532-2

**Published:** 2018-07-28

**Authors:** Motofumi Nakanishi, Shintaro Izumi, Sho Nagayoshi, Hiroshi Kawaguchi, Masahiko Yoshimoto, Toshikazu Shiga, Takafumi Ando, Satoshi Nakae, Chiyoko Usui, Tomoko Aoyama, Shigeho Tanaka

**Affiliations:** 1 Omron Healthcare Co., Ltd., 53 Kunotsubo, Terado-cho, Muko, Kyoto 617-0002 Japan; 2 The Institute of Scientific and Industrial Research Osaka University, 8-1 Mihogaoka, Ibaraki, Osaka 567-0047 Japan; 3 The Graduate School of System Informatics, Kobe University, 1-1 Rokkodai, Nada, Kobe, Hyogo 657-8501 Japan; 4 The Section of Energy Metabolism, Department of Nutrition and Metabolism, National Institute of Health and Nutrition, National Institutes of Biomedical Innovation, Health and Nutrition, 1-23-1 Toyama, Shinjuku, Tokyo 162-8636 Japan; 5 Division of Bioengineering, Graduate School of Engineering Science, Osaka University, 1-3 Machikaneyama, Toyonaka, Osaka 560-8531 Japan; 6 Department of Communication, Division of Human Science, Tokyo Woman’s Christian University, 2-6-1 Zempukuji, Suginami-ku, Tokyo 167-8585 Japan; 7 Department of Nutritional Epidemiology and Shokuiku, National Institute of Health and Nutrition, National Institutes of Biomedical Innovation, Health and Nutrition, 1-23-1 Toyama, Shinjuku, Tokyo 162-8636 Japan

**Keywords:** Energy expenditure estimations, Heart rate, Physical activity, Triaxial acceleration, Physical activity classification, Metabolic equivalents

## Abstract

**Background:**

Herein, an algorithm that can be used in wearable health monitoring devices to estimate metabolic equivalents (METs) based on physical activity intensity data, particularly for certain activities in daily life that make MET estimation difficult.

**Results:**

Energy expenditure data were obtained from 42 volunteers using indirect calorimetry, triaxial accelerations and heart rates. The proposed algorithm used the percentage of heart rate reserve (%HRR) and the acceleration signal from the wearable device to divide the data into a middle-intensity group and a high-intensity group (HIG). The two groups were defined in terms of estimated METs. Evaluation results revealed that the classification accuracy for both groups was higher than 91%. To further facilitate MET estimation, five multiple-regression models using different features were evaluated via leave-one-out cross-validation. Using this approach, all models showed significant improvements in mean absolute percentage error (MAPE) of METs in the HIG, which included stair ascent, and the maximum reduction in MAPE for HIG was 24% compared to the previous model (HJA-750), which demonstrated a 70.7% improvement ratio. The most suitable model for our purpose that utilized heart rate and filtered synthetic acceleration was selected and its estimation error trend was confirmed.

**Conclusion:**

For HIG, the MAPE recalculated by the most suitable model was 10.5%. The improvement ratio was 71.6% as compared to the previous model (HJA-750C). This result was almost identical to that obtained from leave-one-out cross-validation. This proposed algorithm revealed an improvement in estimation accuracy for activities in daily life; in particular, the results included estimated values associated with stair ascent, which has been a difficult activity to evaluate so far.

## Background

Increasing physical activity is particularly important to prevent lifestyle diseases. Accurate monitoring of physical activity intensity (PAI) during daily lifestyle activities has gained popularity. To obtain accurate measurements, the collection of continuous and long-term PAI data is crucial. Coleman et al. [1] found that variation in long-term step monitoring can quantify differences resulting from changes in health status, and that long-term continuous PAI data collection and step counting is an effective method for monitoring and evaluating improvements in lifestyle. Such data are also useful to improve health guidance to facilitate the prevention of lifestyle diseases. Accordingly, a strong ongoing demand exists for devices that can monitor lifestyle activities in terms of PAI data.

Several previous studies have examined specific methods for monitoring PAI data, which have led to the introduction of various devices to collect related information [2–6]. Most of these studies, including our previous work [7, 8] that specifically proposed an estimation algorithm for PAIs for household activities, have explored monitoring devices and algorithms by examining the use of acceleration data [7, 8]. This previous research laid the groundwork for monitoring daily activities using PAI data. However, important shortcomings related specifically to small wearable monitoring devices must still be addressed.

A significant difficulty is associated with the estimation of energy expenditure (EE) for certain activities which cannot be estimated from acceleration data alone, making it difficult to calculate the PAI of such activities accurately. There are two major types of such activities. The first group includes activities with small body movements, but a large PAI, such as cycling or muscle training. The other type includes activities accompanied by an elevation change, such as hiking or stair ascent. The PAIs of these activities tend to be underestimated. An example is ‘stair ascent’, which produced an error rate of − 60.6% in our previous study [8]. Crouter et al. reported that the error rate of one device (ActiGraph Model 7164; ActiGraph LLC, Pensacola, FL) that uses the Freedson MET Equation is − 38.3% for stair ascent and descent [9]. As previously mentioned, monitoring the PAI during daily-life activities with high accuracy is important in the prevention of many lifestyle diseases, and the EE estimation accuracy of these activities must be improved, especially with respect to wearable monitoring devices.

Another major objective of PAI monitoring is to collect data continuously over long periods. Wearable monitoring devices must therefore be suitable for this purpose. When monitoring PAI during daily activities, it will eventually be possible to confirm the results in real time. Hence, wearable devices must independently obtain signals from sensors, process those signals and estimate PAI. Therefore, estimating PAI using a simple approach with minimal cost and power consumption is important if the estimation algorithm is to be integrated into wearable devices.

Various methods have been proposed to improve the PAI accuracy for activities that are difficult to estimate. In previous studies [10–14], the number of PA classifications was increased using multiple sensors and machine learning algorithms such as support vector machine (SVM), and as a result, the accuracy of PAI estimation was improved. For example, Cvetković et al. [10] reported that 30 parameters were obtained from the accelerometer and used in their algorithm. By doing so, the classification numbers and estimation accuracy could be increased. However, the SVM approach is difficult to integrate into wearable devices without support from cloud computing. Therefore, it is apparently not suitable for our purpose because wireless communication with cloud-based analytical software requires a significant amount of additional power consumption.

Another approach is to combine different sensors with an accelerometer [15–23], such as a barometer. The algorithms using a barometer to measure changes in elevation (i.e. altitude) have been reported by Ohtaki and Voleno [18, 19] and others [20, 21]. By improving the estimation formula and classified PA using elevation information, the algorithm can improve the accuracy of EE estimation for stair ascent. However, a barometer typically consumes considerable power, and this method is effective for improving the accuracy of estimating EE for activities that involve a change in elevation, such as stair ascent. However, it does not work for other types of activities such as a cycling because the change in elevation is gradual. Therefore, these conventional methods are not practical for accurate PAI monitoring in daily life, especially in wearable monitoring devices.

Another method uses heart rate data to improve accuracy, as reported by Crouter and Li [22, 23]. A strong correlation exists between PAI and heart rate, and consequently, EE estimation accuracy can be increased. Additionally, the power consumption required to monitor heart rate is very small. For example, Izumi et al. proposed a system-on-a-chip (SoC) sensor which enables heart rate inter-beat interval (RRI) measurements with very low power consumption [24]. Other groups have also proposed low-power-consumption SoCs for measuring heart rate [25, 26]. These studies have confirmed that the marginal increase in power consumption from the addition of a heart rate sensor to a wearable device can be minimized. Furthermore, heart rate data provide valuable biological information related to a person’s health levels, with measurements providing a wide range of useful information beyond PAI estimates, and the addition of a heart rate sensor to a wearable device can thus provide a wide range of benefits. Given this range of benefits, we developed a simple algorithm that combines heart rate and acceleration data.

Crouter et al. reported an error of − 20.5% [22] in the estimation of the EE related to stair ascent by using acceleration and heart rate data. This error is relatively large for actual application. Therefore, this paper addresses the development of the PAI monitoring algorithm, which can improve the estimation accuracy for activities that have previously shown EE estimation difficulty, such as stair ascent. For continuous and long-term monitoring, the proposed algorithm must be embedded in wearable devices. Therefore, we propose a simple algorithm that combines heart rate and acceleration data with a decision tree and multiple regression analysis. The algorithm proposed in this paper is expanded from the algorithm using only accelerations described previously in the literature [7, 8]. The heart rate information is used to resolve difficulties associated with applications that are based solely on acceleration data.

## Methods

This study represents two major improvements over our previous study [8]. First, the number of locomotive activity classification groups has been increased from one to two. Second, the number of parameters used in our multiple-regression models for estimating PAI data has also been increased. We also conducted experiments for the development and evaluation of the estimation algorithms proposed in the present study.

### Signal processing

This paper presents a classification algorithm for increasing the number of classification groups used in locomotive activities. The algorithm uses the following three indices: filtered synthetic acceleration (ACC_fil_), ratio of unfiltered synthetic acceleration to filtered synthetic acceleration (RUF) and the percentage of heart rate reserve (%HRR). This section describes the method used for signal processing of acceleration data and heart rates to calculate these three indices.

#### Triaxial acceleration

The measured triaxial acceleration is processed in a manner similar to that described in our previous study [8]. First, signals from a triaxial accelerometer were run through a high-pass filter with a 0.7 Hz cut-off frequency to remove the gravitational acceleration component, for reasons presented in our earlier report [7]. Fast Fourier transform analysis revealed that for locomotive activities, peak power appeared at a frequency of 1.0 Hz or higher. The peak frequency and walking pace also increased proportionally. For household activities, the peak power appeared at 1.0 Hz or less. The mean frequency of the peak was 0.29 ± 0.19 Hz. The peak value of household activities is strongly influenced by the gravitational acceleration component because of a change in body position. If this influence is removed, the peak value in household activities becomes 1 Hz or higher [7]. Therefore, the cut-off frequency was set as 0.7 Hz (mean + 2SD) to remove the influence of gravitational acceleration on household activities and ensure that acceleration signals during locomotive activities were not affected. Subsequently, the synthetic acceleration along three axes, the anteroposterior axis (X), mediolateral axis (Y) and vertical axis (Z), with the vector magnitude equal to $\sqrt {X^{2} + Y^{2} + Z^{2} }$, was calculated using raw (unfiltered) acceleration signals and the values were then run through a high-pass filter. Finally, the ratio of unfiltered to filtered signals was calculated to classify the activities as household or locomotive activities. ACC_fil_ is defined as the mean value of the synthetic acceleration obtained from the filtered signal during each activity, and is calculated by averaging the mean values of the synthetic acceleration every 10 s.

#### Percentage heart rate reserve (%HRR)

Our methodology uses %HRR, as defined in Eq. (1) below, which was obtained from the available literature [27].

1 $$ \% HRR = \frac{{HR_{act} - HR_{rest} }}{{HR_{\text{max} } - HR_{rest} }} \times 100 $$

The heart rate during activity (HR_act_) represents the mean value of the average heart rate every 10 s during activities, while the heart rate at rest (HR_rest_) is defined as the mean value of the average heart rate over a resting period of 7 min. The maximum heart rate (HR_max_) is calculated based on the Karvonen formula as

2 $$ HR_{\text{max} } = 220 - Age $$

### Estimation algorithm

#### Algorithm for physical activity classification

The first major improvement is to increase the number of classification groups. The previous decision tree classifies the physical activities into three groups, namely sedentary, household and locomotive [8]. In this study, the new node to classify into middle-intensity group (MIG) or high-intensity group (HIG) was appended to the previous decision tree. Sedentary is defined as an activity which has a near-resting energy expenditure, such as sitting. Household activity is defined as a PA excluding locomotion, which nevertheless shows over one MET, such as vacuuming and washing dishes. Although our pioneering study assessed the possibility of increasing the number of classifications within the locomotive activity group [28], this study expanded that concept by further classifying locomotive activities into MIG and HIG activities. These groups were classified using %HRR in the proposed decision tree to utilize the correlation between PAI and %HRR.

**Fig. 1:**
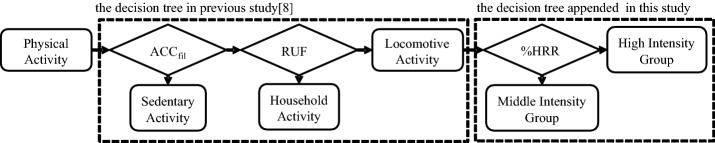
Proposed decision tree for the new algorithm

We define MIG activities as activities involving six or less METs, such as walking at a normal speed. HIG activities are defined as activities involving more than six METs, such as jogging and stair ascent. This value (six METs) reflects a cut-off value in the guidelines established by the American College of Sports Medicine [29]. Figure 1 shows the decision tree used for our PA group classifications.

A previous study divided locomotive activity from other activity groups used by ACC_fil_ and RUF. Locomotive activity is divided into two additional groups by %HRR.

#### Estimation of METs as EE

The second improvement involves an increase in the number of independent variables used in the multiple-regression model for PAI estimation. This paper proposes five multiple-regression models using software (SPSS Statistics 24, SPSS24; IBM Corp., Armonk, NY). The forced entry method was employed to confirm the influence of quantities of certain parameters used in the respective regression models to obtain the estimation results. Table 1 presents the parameters used in each of the proposed models. In this study, four parameters were selected, including acceleration, %HRR, body mass index (BMI) and weight. BMI was used not only as the weight value but also as a characteristic representing body shape.

**Table 1:** List of features for each proposed model

Model	Features
Prop. 01	ACC_fil_
Prop. 02	HRR
Prop. 03	ACC_fil_, HRR
Prop. 04	ACC_fil_, HRR, BMI
Prop. 05	ACC_fil_, HRR, weight
Previous	ACC_fil_

The accuracies of the proposed models were compared based on the mean absolute percentage error (MAPE) of estimated MET values produced by the leave-one-out cross-validation. In this process, one part of the data observation serves as the validation set and the other serves as the training set. The cross-validation process was then repeated for a number of subjects, with each subject used only once to obtain the validation set. The results from the respective subjects were averaged to produce a single estimate. Based on a comparison of the results, the most suitable multiple-regression model was selected for our study.

### Experimental methods

**Fig. 2:**
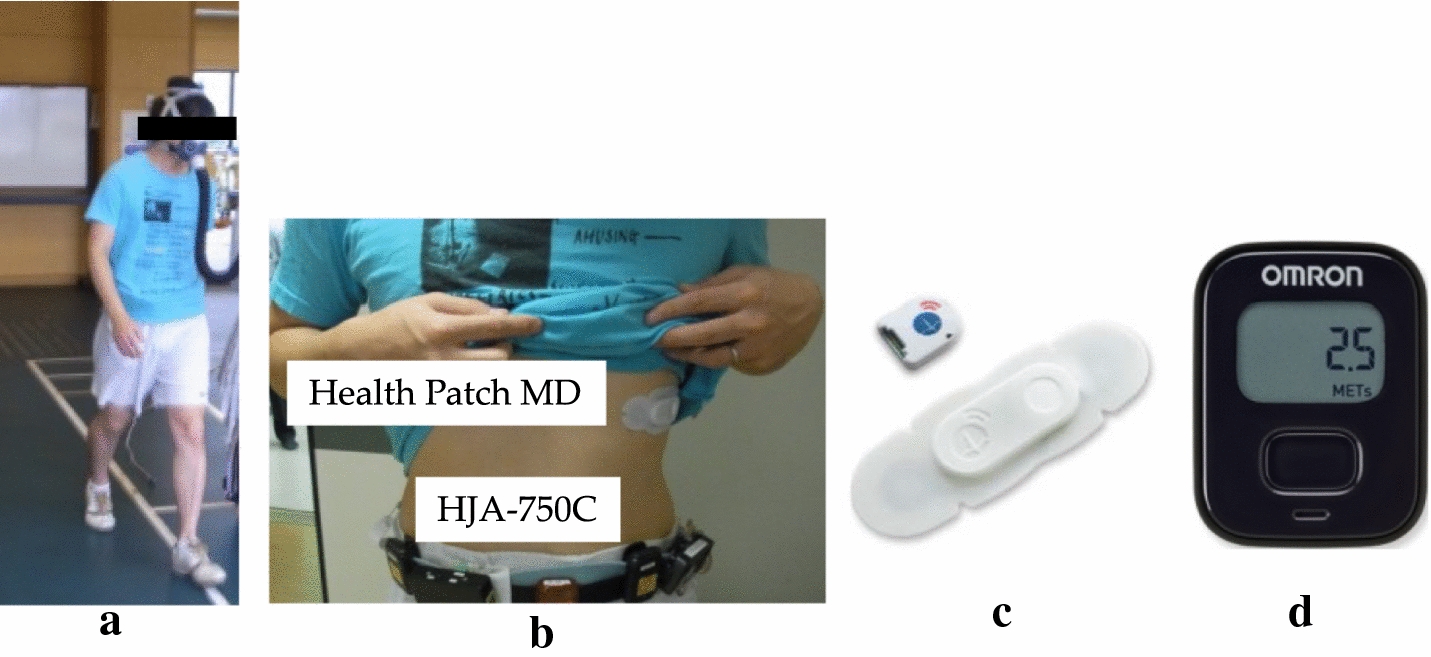
**a** Subject during measurement. **b** Location of Health Patch MD and HJA-750C. **c** Health Patch MD. **d** Activity monitor (HJA-750C)

To develop and evaluate the proposed classification algorithm and the EE estimation model, we measured the METs, triaxial acceleration and RRIs of volunteer test subjects as they performed various activities (Fig. 2a).

#### Subjects

A total of 42 volunteers participated in the experiments for this study, which were conducted at the National Institute of Health and Nutrition (NIHN) in Tokyo, Japan, following the guidelines laid down in the Declaration of Helsinki. All procedures involving human subjects were approved by the Ethical Committees of NIHN and by Omron Healthcare Co., Ltd. Subjects were excluded from the study if they showed any contraindication to exercise, or if they were physically unable to complete an activity. Approximately five subjects were chosen from each 10-year age range and gender (Table 2).

**Table 2:** Physical characteristics of subjects (*N* = 42)

Age groups	N	Age (years)	Height (cm)	Weight (kg)	BMI (kg/m^2^)
		Avg. (SD)	Avg. (SD)	Avg. (SD)	Avg. (SD)
Male					
20–30	6	26.2 (3.1)	169.0 (7.2)	66.3 (10.6)	23.1 (2.0)
30–40	4	36.3 (2.8)	171.1 (3.1)	65.6 (15.2)	22.3 (4.4)
40–50	6	43.2 (3.9)	173.1 (6.2)	73.1 (12.1)	24.4 (3.6)
50–60	5	52.2 (1.8)	171.5 (2.3)	68.4 (13.1)	23.2 (3.9)
Female					
20–30	5	23.0 (2.3)	157.3 (4.5)	49.1 (5.1)	19.8 (1.5)
30–40	6	32.5 (3.4)	163.1 (11.0)	59.0 (14.9)	22.0 (4.2)
40–50	5	43.0 (4.2)	155.9 (5.7)	52.8 (17.1)	21.6 (6.4)
50–60	5	52.8 (1.3)	158.1 (2.2)	59.4 (8.0)	23.8 (3.2)

The weights and heights of all subjects were measured to produce a BMI for each. The age, gender, height, weight and BMI distributions of the test subjects are listed in Table 2. Details related to the purposes and procedures involved in this study were explained to subjects before measurements were taken, with prior written informed consent obtained from all subjects.

#### Experimental setup for data measurement

The study required the monitoring of triaxial acceleration, RRI and EE for various physical activities conducted for a set period. Test subjects performed 23 distinct activities (including resting in a seated position), during which their triaxial acceleration and RRI values were recorded using the Health Patch MD function (Vital Connect Inc., San Jose, Ca). In this study, heart beats per minute were converted from those recorded RRI by Health Patch MD and used to calculate %HRR; it was developed for 24-h monitoring. A clinical validation of this device has been provided in a previous report [30], and an activity monitor (HJA-750C; Omron Healthcare Co., Ltd., Kyoto, Japan) was positioned at each subject’s waist. Figure 2b shows the location of these devices.

During each activity, the subjects’ exhaled respiration was collected in a Douglas bag. The EE values were estimated from volumes of oxygen and carbon dioxide, reported as VO_2_ and VCO_2_, respectively, using Weir’s equation [31]. For reference, the MET values were calculated by dividing the EE during the activities by the measured resting metabolic rate. This study specifically addressed eight activities, including stair ascent/descent, walking (three speeds), walking with load (two patterns) and jogging, all of which were identified as locomotive activities. These experiments were conducted in a controlled laboratory setting. The HR_rest_ utilized average heart rate data, after excluding the first 3 min, for 10 min of sitting. For six of the activities (excluding stair ascent and descent), the subjects were instructed to walk at a speed determined using a pace leader. Stair ascent and descent were evaluated with the subjects selecting their own speed. Table 3 shows the speed and time for each of the eight activities.

**Table 3:** Eight locomotive activities evaluated in this paper

Activity	Speed	Time (min)
Stair descent	Self-selected	2.5
Stair ascent	Self-selected	2
Slow walking	55 m/min	5
Normal walking	70 m/min	5
Brisk walking	100 m/min	5
Normal walking with load (3 kg)	70 m/min	5
Slow walking with load (5 kg)	55 m/min	5
Jogging	130 m/min	4

## Results

### Measurement results

Table 4 shows averages and standard deviations (SDs) of METs and %HRR for each activity; the respective numbers of test subjects for the eight activities are also listed. Measurements for two of the subjects could not be conducted for any activity. Measurement failures occurred during a number of activities for several subjects; these results were excluded from the study. In stair ascent and jogging activities, MET values of more than six were defined as HIG activities. Because the other six activities always have MET values less than six, they were defined as MIG activities.

**Table 4:** %HRR and measured METs for each activity

Activity	N	%HRR [%]		METs	
		Avg.	SD	Avg.	SD
MIG					
Stair descent	36	15.45	7.11	2.73	0.39
Slow walking (55 m/min)	33	18.81	9.10	3.35	0.54
Normal walking (70 m/min)	29	23.37	9.14	3.75	0.50
Brisk walking (100 m/min)	30	34.16	11.87	5.12	0.88
Slow walking with Load (5 kg)	33	27.01	9.50	4.03	0.46
Normal walking with Load (3 kg)	29	26.92	9.99	4.24	0.65
HIG					
Stair ascent	30	51.22	8.52	7.42	0.87
Jogging (130 m/min)	23	67.34	15.37	9.50	1.64

**Fig. 3:**
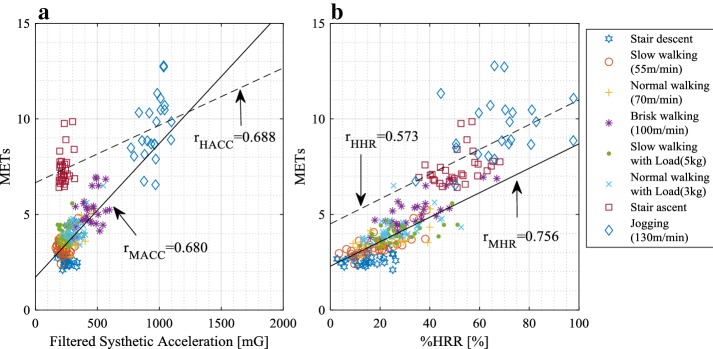
Measurement results from the eight activities. **a** Relation between ACC_fil_ and METs. r_HACC_ is the correlation coefficient of the relation between ACC_fil_ and METs in HIG, and r_MACC_ is the correlation coefficient of MIG. **b** Relation between HRR and METs. The correlation coefficient of the relation between %HRR and METs is r_HHR_ for HIG and r_MHR_ for MIG

The ACC_fil_ and %HRR measurement results for the eight locomotive activities are presented in Fig. 3. Table 5 presents the statistical results of the METs, ACC_fil_ and %HRR analyses. The three indexes were analysed by using linear mixed effect model with SPSS statistics 24. The all indexes have significant differences between MIG and HIG (pairwise comparisons, p < 0.05). Figure 3a shows the relation between MET and ACC_fil_ results for each activity. In MIG, the mean − 3SD of ACC_fil_ was 25.3 [mG] and the mean + 3SD was 562.8 [mG]. The stair ascent results followed the same distribution trends as those reported in our previous study [8]. The distribution of the latter is depicted in Fig. 3a.

**Table 5:** Statistical results of measurement indices

Index	*p* value	MIG (n = 190)		HIG (n = 53)	
		Avg.	SD	Avg.	SD
ACC_fil_ [mG]	< 0.001	294.1	89.6	545.8	367.9
%HRR [%]	< 0.001	23.96	11.20	58.2	14.3
METs	< 0.001	3.83	0.95	8.3	1.6

The p-value obtained from the pairwise comparisons between MIG and HIG of each index by using linear mixed effect model

The ACC_fil_ of MIG correlates with the METs (*r* = 0.680), as the ACC_fil_ of HIG does (*r* = 0.688). Figure 3b presents the relation between METs and %HRR. The mean + 3SD of the %HRR for HIG was 102.15%, whereas the mean − 3SD of %HRR for HIG was 13.11%. The %HRR result of HIG activity had a relatively large SD (= 14.34), as shown in Table 5. However, the HIG average %HRR was more than twice as large as the average %HRR for MIG. Additionally, there were two distributions separated at around 40%, and correlation existed between %HRR and METs of the MIG (*r* = 0.756) and HIG (*r* = 0.573).

#### Classification and MET Estimation Result

**Fig. 4:**
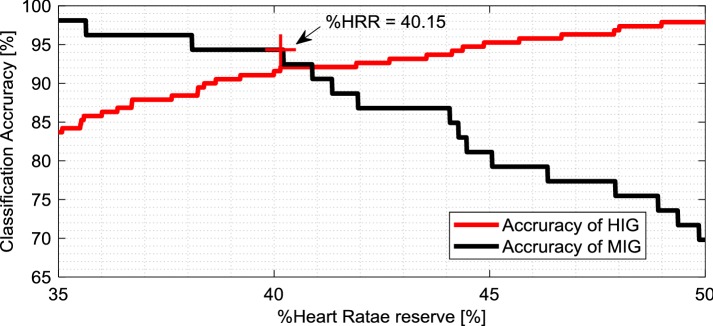
Relation between classification accuracy of MIG and HIG and %HRR

We used a decision tree to further classify the locomotive activity into two groups according to %HRR. For PA classification, this study first identified the %HRR values for which the classification accuracy of MIG and HIG was the highest. Figure 4 shows the relation between %HRR and the classification accuracy of MIG and HIG. As a result, the %HRR with the highest observed classification accuracy was 40.15%. This was close to the boundary value between Light and Moderate (%HRR = 40%) for classifying activities by %HRR established in the ACSM guidelines. For this reason and for simplification, the threshold for HIG and MIG was set at 40% instead of 40.15%.

Table 6 shows the classification accuracy for each activity. For activities in MIG, 91.6% were correctly classified. For activities in HIG, 94.3% were correctly classified, while 5.7% were misclassified into MIG.

**Table 6:** Results of group classification using the proposed decision tree

	Percentage of classified result	
	Classified as MIG [%]	Classified as HIG [%]
MIG	91.6	8.4
Stair descent	100.0	0.0
Slow walking (55 m/min)	100.0	0.0
Normal walking (70 m/min)	96.6	3.4
Brisk walking (100 m/min)	76.7	23.3
Slow walking with load (5 kg)	87.9	12.1
Normal walking with load (3 kg)	86.2	13.8
HIG	5.7	94.3
Stair ascent	6.7	93.3
Jogging (130 m/min)	4.3	95.7

As described in “Estimation of METs as EE” section, validation results were produced by the leave-one-out cross-validation method. This approach involves utilizing one part of the observed data as the validation set and the remaining observations as the training set. The parameters used for each model are shown in Table 1. Table 7 shows the average MAPE values for the respective processes. First, we focused on the examination of HIG. The results showed no clear differences for Props. 03, 04 and 05. By contrast, the MAPE produced by Props. 01 and 02, which used only acceleration or %HRR, were 1.3–3.0% larger, respectively, than those of the other three models associated with HIG. As a result of comparison with the previous algorithm (HJA-750), Prop. 05 was the most improved, and its value was the result of reducing the MAPE by 26.1%. This result, therefore, indicated a significant improvement. Next, attention was paid to stair ascent, which is difficult to estimate. All models showed great improvement and Prop. 03 provided the lowest MAPE at 10.0%, for stair ascent. In addition, examination of MIG indicated that Prop. 03 with a MAPE of 15.3% performed better than the other models. In addition, the errors of the proposed models were almost the same as those found for the previous model (HJA-750C).

**Table 7:** MAPE of respective estimated results [%]

	Proposed multiple-regression models					HJA-750C	
Activity	Prop. 01	Prop. 02	Prop. 03	Prop. 04	Prop. 05	N	
Stair descent	31.6	22.9	21.6	22.0	22.3	36	28.6
Slow walking (55 m/min)	12.1	11.1	8.8	9.0	8.8	33	13.2
Normal walking (70 m/min)	13.6	11.5	12.1	12.6	12.4	29	11.9
Brisk walking (100 m/min)	18.7	17.9	15.9	17.4	16.8	30	11.8
Slow walking with load (5 kg)	18.6	15.8	16.2	17.0	16.4	33	15.8
Normal walking with load (3 kg)	17.5	17.0	16.7	17.7	16.9	29	10.5
Stair ascent	11.7	13.5	10.0	10.5	10.5	27	58.7
Jogging (130 m/min)	13.5	14.7	12.8	12.4	11.8	23	11.4
MIG	19.0	16.2	15.3	16.1	15.7	189	15.8
HIG	12.4	13.8	11.1	11.1	10.8	50	36.9

The MAPE for each PA was calculated for the five proposed models. Prop. 01 used only ACC_fil_, Prop. 02 used only %HRR, Prop. 03 used both ACC_fil_ and %HRR, Prop. 04 added BMI to Prop. 03 and Prop. 05 added weight to Prop. 03. The HJA-750C results were also presented for comparison. HJA-750C estimated METs using the earlier reported algorithm, which used acceleration and treated the MIG and HIG as one group with regard to locomotive activity, where N is the number of subjects.

Based on the results of the leave-one-out cross-validation, Prop 3 should be selected as the most suitable model for the purpose of this paper. Because the number of features used by Prop.3 is the smallest and the calculation amount can be made small, although there was no clear difference between the MAPE values for Props. 04 and 05. This paper selected Prop. 03 results to confirm the error trends, recalculating the multiple-regression models for MIG and HIG using all measured data as the training data. The resulting regression equations for MIG and HIG are shown, respectively, in Eqs. (3) and (4) below.

3 $$ MIG{:}METs_{est} = 0.0043 \cdot ACC_{fil} + 0.047 \cdot \% HRR + 1.4238 $$

4 $$ \text{HIG}{:}METs_{est} = 0.0024 \cdot ACC_{fil} + 0.029 \cdot \% HRR + 5.3113 $$

**Fig. 5:**
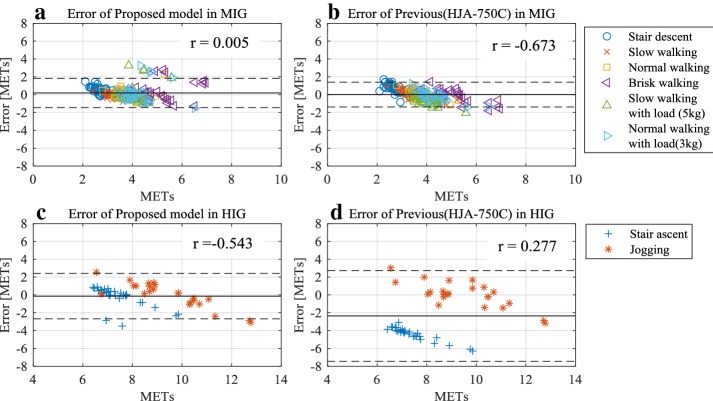
METs and error rates obtained using the proposed model and the algorithm reported earlier: **a** MIG relation with the proposed model, **b** MIG result from the previous model, **c** HIG relation with the proposed model and **d** HIG result from the previous model

Figure 5 shows the relation between the estimation error and the METs, which was estimated using Prop. 03 and the previous model (HJA-750). The solid lines depict the mean values of MIG and HIG, while the dashed lines represent 95% prediction intervals (PI) of error. Table 8 presents the statistical results of the estimation error. The MIG result using the proposed model had a few fixed biases because the 95% confidence interval (CI) of the average ranged from 0.07 to 0.31. However, there was no proportional error (*r* = 0.005). The HIG results obtained using the proposed model showed no fixed bias because the 95% CI of the average ranged from − 0.48 to 0.21. However, there was a proportional error (*r* = − 0.543). Some stair ascent results showed errors that were less than − 3.0 METs, although all errors corresponding to the results of the previous model were less than − 3.0 METs.

**Table 8:** Statistical results of estimation based on all data

	MIG		HIG	
	Proposed	Previous	Proposed	Previous
Average	0.19	0.01	− 0.14	− 2.35
Upper confidence interval of the average (95%)	0.31	0.11	0.21	− 1.64
Lower confidence interval of the average (95%)	0.07	− 0.09	− 0.48	− 3.06
Upper prediction interval (95%)	1.84	1.40	2.41	2.73
Lower prediction interval (95%)	− 1.46	− 1.38	− 2.68	− 7.44

The results obtained using our previous model and the proposed model is compared in Table 9. The MAPE of MIG and HIG in Table 9 were calculated from all data defined in their groups. The results showed that the MAPE and MPE using the proposed model was improved compared to the previous algorithm for the MIG and HIG activities; especially, the MAPE for HIG improved by 26.45% (= 36.95–10.50). It was confirmed whether there was a significant difference in MPE as the error distribution between proposed and previous results with Wilcoxon signed-rank test by using SPSS statistics 24. In three activities including stair ascent, there were significant differences (p < 0.05) in the MPE values associated with results in this study and our previous results.

**Table 9:** MAPE and MPE for the eight activities

Activity	MAPE [%]		MPE [%]		
	Prop.	Prev.	Prop.	Prev.	p-value
Stair descent	21.22	28.56	20.02	26.63	0.004
Slow walking	8.19	13.2	− 0.04	5.06	0.810
Normal walking	11.48	11.88	2.65	3.94	0.381
Brisk walking	15.59	11.79	4.27	− 2.15	0.614
Slow walking with load (5 kg)	15.82	15.84	1.44	− 15	< 0.001
Normal walking with load(3 kg)	16.23	7.47	4.31	− 1.87	0.873
Stair ascent	9.61	58.70	– 2.24	− 58.70	< 0.001
Jogging	11.66	11.41	2.1	2.47	0.927
MIG	14.88	15.31	5.77	3.32	0.611
HIG	10.50	36.95	− 0.36	− 30.56	< 0.001

The p-values were obtained by Wilcoxon signed-rank test for each activity between proposed and previous in MPE. There was a significant difference in three activities in MPE (p < 0.05)

**Fig. 6:**
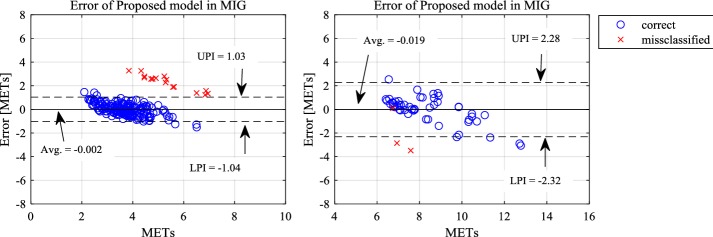
Relation between the error and the result of classification. The straight line shows the average value with correct classifications, while dashed lines represent 95% prediction interval. UPI and LPI are the upper and lower prediction intervals, respectively

Additionally, we confirmed the error distribution based on the classification results presented in Fig. 6. The PI of the correct classification data for the MIG ranged from − 1.036 to 1.033 METs, although the PI of the HIG ranged from − 2.32 to 2.28 METs. Large errors were found for both groups when the results were incorrectly classified. The MIG errors had a mean value of 2.29 METs, whereas the HIG errors had a mean of approximately − 3 METs.

In this paper, MAPE (i.e. the estimation accuracy) was compared with other algorithms using a random forest and REPTree. These are machine learning methods which use decision trees. The results are shown in Table 10. Although the data used for estimation and the number of activities were different, their results showed no clear differences between the proposed and others. The result of Luštrek [11] is better than proposed result. However, as described in Table 9, the MAPE of jogging using the proposed model is 11.66% and can be confirmed to be lower than the result of Luštrek [11].

**Table 10:** Mean absolute percentage error compared with other algorithms

Author	Algorithm	Refs.	Number of features	Activity	MAPE [%]
Proposed	Classification tree Multi-regression model		2	6 different walks, jogging, stair	13.9
Mitja Luštrek	REPTree	[11]	8 (accelerometer)	Running	12.6^a^
Hristija Gjoreski	Random forest Multiple Contexts Ensemble	[12]	128 (accelerometer)	Walk, running	15.7

^a^This value was calculated as the MAPE to the mean value of METs at two different speeds

In addition, the estimation accuracy during stair ascent was compared with other algorithms, as an example of activities difficult to estimate. Table 11 lists the results compared with those obtained using other algorithms, which indicates that the proposed model has an advantage over other estimation methods, excluding the method of Wang. Note that the proposed method was realized with fewer features, and the difference in accuracy was only 0.3%.

**Table 11:** MPE in stair ascent with other algorithms

Algorithm	Refs.	Features	Activity	MPE [%]
Proposed model		Accelerometer, %HRR	Stair ascent/descent	9.90
ActiGraph new 2-regression model	[9]	Count	Stair ascent/descent	− 11.76
Actiheart combined activity and HR algorithm	[22]	Count^a^ HR	Stair ascent/descent	− 20.51
Proposed model		Accelerometer, %HRR	Stair ascent	− 2.24
Matteo Voleno	[19]	Accelerometer, barometer	Stair ascent	6.6
Jinging Wang	[32]	21 features (form accelerometer and barometer)	Stair ascent	− 1.96

^a^‘Count’ is the index calculated from acceleration using ActiGraph

## Discussion

This paper proposes an algorithm to estimate METs for daily-life activities, including those for which MET estimation is difficult, such as stair ascent. Table 9 shows the MAPE and MPE of the respective activities. The proposed algorithm achieved higher accuracy in both MIG and HIG compared to the previous algorithm available in the commercial product HJA-750. MAPE decreased by 0.43% in MIG and 26.45% in HIG, in comparison with the previous model. Specifically, the estimation accuracy of stair ascent activity was clearly increased. In terms of the stair ascent activity, MAPE was 9.61%. However, the MAPE of METs with the previous model (HJA-750C) was 58.70%. The MAPE of the proposed model was estimated to be 49.09% smaller than that of the previous model, which is an improvement of approximately 84%. These results demonstrate the superiority of the proposed model for accurate MET estimation for HIG, including that for the stair ascent activity.

We confirmed the error derived from comparing the proposed model and our previous model (HJA-750C) for MET estimation. The relation between METs and error rates was confirmed when all data were used as training data. Figure 5c, d show the relation between the error rate and METs for HIG. These figures clarify that the use of the proposed model results in a significant improvement over the previous model with respect to estimation accuracy. This improvement is supported by the data in Table 8, where 95% PI of the proposed model ranged from − 2.68 to 2.41, whereas the PI obtained using the previous model ranged from − 7.44 to 2.73. These results indicated a clear improvement over the previously used algorithm.

The results obtained in this study were evaluated through comparisons with previous algorithms [9, 11, 12, 19, 22, 32]. Table 10 shows the results of our comparison with other algorithms using machine learning. There were no clear differences using the proposed model. Although it is a comparison with different datasets, it was possible to obtain an estimation accuracy similar to that of machine learning methods, only we accomplished this with a very simple algorithm. These results strongly suggest that the proposed algorithm represents a significant improvement over other versions and methods. Table 11 presents the MPE results for stair ascent and descent. An improvement in accuracy of about 10% was observed compared to the result of the Actiheart combined activity and the HR algorithm, and the MPE of the proposed model was lower by about 2% for the ActiGraph 2-regression model. Therefore, the proposed model demonstrates better estimation accuracy than other algorithms that use acceleration and heart rate data.

Similar results were found for the MPE in stair ascent compared to other algorithms that use an accelerometer and a barometer. The result reported by Voleno et al. [19] was 6.6%. The MPE of the proposed model was lower. The algorithm reported by Wang et al. [32] was slightly better than our proposed model, by about 0.3%. According to Table 4, the METs during stair ascent were about seven and the difference between the proposed model and Wang’s [32] amounted to about 0.08. Thus, there was a very little difference between the proposed algorithm and Wang’s algorithm [32]. In addition, Wang’s algorithm [32] used 13 parameters, while our proposed algorithm used only two parameters and a simple decision tree. Therefore, the processing overhead required by our proposed algorithm was less than that put forward by Wang [32]. In addition to these advantages, our proposed algorithm can be implemented with relatively low power consumption.

Considering the causes of error, this study confirmed the relation between error rate and classification results. As depicted in Fig. 6, a large error rate in MIG (over two METs) was traceable to misclassification issues. Assuming that these can be eliminated, the revised 95% prediction interval would be − 1.04 to 1.03, which is better than that obtained with HJA-750C (− 1.38 to 1.40). Therefore, it is clear that the most effective means of improving error rates using the proposed model is to suppress misclassification. To achieve this, it is necessary to revise the equation of %HRR calculation and to add individual adjustments.

The result of the classification accuracy is described in Table 6. The average classification accuracy was higher than 91% for both MIG and HIG. Thus, one can reasonably conclude that our proposed decision tree is appropriate. However, the classification result of brisk walking was relatively low (76.7%) compared to those of other activities. The results showed that the %HRR trends for misclassified subjects differed from other results that were classified correctly. To ascertain the cause of these %HRR trend differences, physical information related to misclassification of subjects who were walking briskly was examined more closely. It was found that five out of the seven misclassified subjects were female, even though the male/female ratio for all test subjects was 50%. These results suggest that sex has a strong effect on %HRR.

To address this issue, it is necessary to consider sex %HRR calculation, as reported by Whyte et al. who used equations for calculating the maximum heart rate that considered sex and age [33]. For example, in the case of sedentary females, Eq. 5 below was used.

5 $$ HR_{\text{max} } \left( {Sedentary\;Female} \right) = 221 - 1.09 \cdot Age $$

The adoption of this equation makes it possible to improve the maximum heart rate accuracy estimation utilized in %HRR calculation.

In addition, subjects with a BMI of over 25 were 30% of all subjects, while those with a BMI over 25 (for brisk walking) were misclassified 57% of the time. This result suggests that BMI also affects %HRR accuracy and that adjusting %HRR to reflect individual BMI values would also improve the classification accuracy.

Another key to improving %HRR is adjusting it to reflect an individual’s resting heart rate. More specifically, because our algorithm can classify resting intervals, %HRR can be recalculated and adjusted using the heart rate obtained while the individual is resting, making it possible to improve the overall classification accuracy. In our experiments, %HRR was obtained from the heart rate obtained in a resting state (i.e. sitting position), which can be frequently observed. Therefore, we believe that setting and recalibrating the resting heart rate from the daily measurement of heart rate can further improve the calculation of %HRR.

In this study, as a first attempt to develop an EE estimation algorithm for daily life activities, the proposed algorithm was developed and evaluated using a dataset which is the measurement results of limited activities conducted by 42 subjects in a controlled laboratory setting. Although the data are limited by the number of subjects, these results confirmed that the proposed model is useful to estimate the METs of high-intensity activities in daily life, including those for which it is difficult to accurately estimate EE using a wearable device. This paper focuses on stair ascent as an activity which is difficult to estimate, and the proposed algorithm can be expected to improve accuracy in other difficult-to-estimate activities, such as cycling and muscle training. The proposed algorithm realized concurrent recording of %HRR, acceleration and PAI more accurately than previously used methods. Hence, the proposed algorithm appears to be very promising for future health guidance.

## Conclusions

We proposed an algorithm which can estimate METs as physical activity intensity data, especially during high-intensity activities in daily life, including stair ascent. The results of our study are supported by two major improvements. First, we used %HRR to classify locomotive activities into two more groups using decision trees. Furthermore, we proposed five multiple-regression models and selected the most suitable one for our purposes based on leave-one-out cross-validation. As a result, the proposed model showed a MAPE of 10.5%, which was 26.45% smaller than that of the previous model, or an improvement of approximately 72%—an 84% reduction in the mean absolute percentage error for stair ascent compared to an earlier model (HJA-750C). Also, the MPE was improved by about 10% compared to the other algorithm that also combined heart rate and acceleration. These results indicate that the proposed algorithm can estimate METs with improved accuracy during daily-life activities including those that are difficult to estimate, such as stair ascent.
